# Global image of countries in international wars: A scoping review of influencing factors

**DOI:** 10.1371/journal.pone.0334095

**Published:** 2025-10-08

**Authors:** Dongyong Li, Kim Hua Tan, Jamsari Alias, Norazila Mat

**Affiliations:** 1 Faculty of Social & Leisure Management, Taylor’s University, Malaysia; 2 Pusat Pengajian Citra Universiti & Institut Islam Hadhari, Universiti Kebangsaan Malaysia,; 3 Faculty of Economics and Management, Universiti Kebangsaan Malaysia; NingboTech University, CHINA

## Abstract

This scoping review investigates factors influencing the international or global image of countries involved in international wars. Following Arksey and O’Malley’s 5-step framework, key sources for the search include academic databases such as Google Scholar, Scopus, and Web of Science. Reporting followed the PRISMA extension for Scoping Reviews (PRISMA-ScR) (Peters et al., 2020) and was guided by the Joanna Briggs Institute (JBI) methodological guidelines. We systematically analyzed 56 peer-reviewed articles published between 2015 and 2024. Our analysis reveals that a country’s global image during war is predominantly shaped by the dynamic interplay of three core factors: (1) Historical factors, providing cognitive and affective frameworks for interpreting conflict; (2) Diplomatic factors, managing critical narrative alignment and credibility constraints; and (3) Leadership factors, embodying national resolve through symbolic representation and communicative authenticity. These factors function as interdependent pillars, where historical narratives constrain diplomatic choices, leadership enacts diplomatic narratives, and diplomacy mediates historical burdens. While secondary factors (e.g., military conduct, cultural proximity) play a role, they are largely interpreted through these core mechanisms. Additionally, the review highlights the role of emerging factors—including digital technology, affective investments, subliminal priming, diversity of information sources, and visual tropes—which represent evolving mechanisms of image formation in contemporary wars. There is a surge in research coinciding with major contemporary wars (e.g., Russo-Ukrainian War). Crucially, this interdependence underscores the necessity for integrated statecraft that strategically aligns historical reframing, diplomatic consistency, and leadership authenticity. However, the efficacy of these core factors is significantly mediated and amplified by secondary factors—such as military conduct, governmental credibility, cultural proximity, and geopolitical positioning—which collectively shape the perception and credibility of a nation’s projected image. Furthermore, emerging factorsintroduce new complexities into the image formation process, accelerating the spread and emotional resonance of narratives while also increasing scrutiny of state actions. These findings offer critical insights for policymakers, media professionals, and scholars seeking to understand and manage a nation’s global standing during and beyond conflict, moving beyond media-centric explanations of wartime perception.

## Introduction

The global image of a country is a complex and multifaceted construct that can have a significant influence on its diplomatic relations, economic opportunities, and global standing [1]. In the context of international wars, this image becomes increasingly critical, as it can shape the perceptions of allies, adversaries, and the international community at large [2]. Perceptions of a country during and after the war have extensive implications, which range from humanitarian aid to trade agreements and political support, among others [3–5].

The shift from print to digital war reporting has transformed report presentation through instant updates and wider accessibility, while fundamentally altering audience engagement patterns [6]. Social media, in particular, has emerged as a powerful tool for governments and individuals to share information in real-time and shape their global image [7]. Image theory offers new perspectives in understanding how the media shapes public perceptions of war [8]. Media representations, such as how wars are reported and wars are portrayed through specific narratives that emphasize heroism, tragedy, or the impact on civilian life, which greatly influence how audiences perceive countries at war [9,10].

While the impact of media factors on the global image of the warring states has attracted scholarly attention, understanding other factors influencing the global image of states involved in international wars is of key importance for policymakers, media professionals, and scholars alike [11–13].

This review aims to investigate the factors that contribute to the formation and evolution of a country’s global image during an international war, focusing on the period between 2015 and 2024. By examining the factors that influence a country’s global image during international war, this review aims to provide an overview of the current state of research and to identify gaps that can be addressed by future research.

Establishing research objectives forms the foundation for research success and directs and motivates during the process. This ensures that the research work is conducted correctly to yield the desired results and outcomes. Thus, the objective of this review is to identify and analyze those key factors influencing a Country’s global image in the context of international wars.

## Methodology

We conducted a scoping review to gain a comprehensive understanding of the factors influencing global image and its research trends. Generally, a scoping review is an appropriate method for assessing the breadth and depth of literature on a specific topic [14]. As a valuable precursor to systematic reviews, scoping reviews are exploratory and descriptive. They aim to identify and map the overall landscape of existing literature, address overarching review questions, elucidate key concepts, and pinpoint knowledge gaps within the research field [15,16].

To systematize the research methodology, we employed the approach proposed by Arksey and O’Malley’s 5-step framework [17]. This includes: (1) identifying the research question; (2) identifying relevant studies; (3) study selection; (4) charting the data; and (5) collating, summarizing, and reporting the results. Additionally, we adopted the Preferred Reporting Items for Systematic reviews and Meta-Analyses extension for Scoping Reviews (PRISMA-ScR) framework as suggested by Peters et al(2020) [14].

### Application of frameworks to achieve research objectives

To ensure the scoping review process systematically addressed the research objective of identifying and analyzing key factors influencing a country’s global image in the context of international war, we applied the framework of Arksey and O’Malley and PRISMA-ScR as follows:

### Identifying the review research question (Step 1)

In this section, we focus on the purpose of this study. The first step in our review is to revisit the research question. Clarifying the research question serves as a standard for analysis, guiding the entire study and providing a critical basis for interpreting this review.

Our review question included the following

***What factors influence the global image of countries involved in international wars?***

***How do these factors shape the global image of these countries?***

This review outlines the factors that influenced the shaping of the country’s global image during the war. We have derived specific factors from past research.

### Identification of relevant studies (Step 2)

After defining the research question, objectives, and scope, we formulated strategies for selecting search keywords/phrases, sources/databases, language, and time span. We conducted comprehensive and systematic searches across multiple major academic databases to ensure broad coverage and minimize selection bias. The selected databases encompassed:

Multidisciplinary Citation Indices: Web of Science Core Collection (searches were conducted using the Web of Science Core Collection via the *www.webofscience.com* interface provided by Clarivate) and Scopus (searches were conducted using the Scopus database via the *www.scopus.com* interface provided by Elsevier.). These are widely recognized as the most comprehensive and authoritative sources for high-impact, peer-reviewed international literature across diverse disciplines. Their rigorous indexing and citation tracking capabilities are considered the gold standard for systematic evidence synthesis [18,19].

Complementary Broad Search Engine: Google Scholar (searches were conducted directly via *scholar.google.com*). While less structured than the indexed databases, Google Scholar is valuable for its ability to retrieve publications from lesser-known journals or repositories that might be missed by traditional databases, thus enhancing the comprehensiveness of our search [20].

This combination of multidisciplinary citation indices (WoS, Scopus), and a broad complementary search engine (Google Scholar) is a well-established strategy recommended in scoping and systematic review methodology literature to balance comprehensiveness, relevance, and feasibility [21]. It aims to capture the breadth of potentially relevant literature while leveraging the strengths of different search platforms.

We searched using inductive search strings, focusing on topic relevance. As shown in Table 1, we improved the accuracy and reliability of the data by using the Boolean operators “OR” and “AND” [22]. Additionally, we limited this study to English-language research from 2015 to 2024 and academic articles from peer-reviewed journals (including research articles, reviews, and conference papers published in journals). This focus is justified because peer-reviewed journals represent the primary channel for disseminating validated, high-quality scholarly research. The peer-review process provides a crucial mechanism for ensuring methodological rigor, credibility, and the reliability of findings, which is essential for synthesizing robust evidence in a scoping review [23,24]. While recognizing that other sources (e.g., books, reports, theses) may contain relevant insights, the sheer volume and core nature of peer-reviewed journal articles make them the most efficient and reliable source for addressing our research objectives and mapping the current state of academic knowledge on this topic within feasible resource constraints [25].

**Table 1:** Summary of Search Terms.

Search Engine	Limiters	Search String	Results
Google Scholar	Articles from 2015–2024	Boolean operators: (“international image” OR “country image” OR “reputation image” OR “global image”) AND (“war” OR “warfare” OR “international war”)	1,000
Scopus	Articles from 2015–2024	Boolean operators: (“international image” OR “country image” OR “reputation image” OR “global image”) AND (“war” OR “warfare” OR “international war”)	69
Web of Science	Articles from 2015–2024	Boolean operators: (“international image” OR “country image” OR “reputation image” OR “global image”) AND (“war” OR “warfare” OR “international war”)	63

We also considered commonly used terms such as “perception” and “reputation” in our initial search. However, using these terms failed to identify a large number of new relevant studies. When we used the term “image”, we could also retrieve terms like “reputation”; therefore, to keep the search focused and manageable, we finally decided to use “image” as the core search term.

After searching and screening the databases, a total of 1,132 articles were collected and exported in ENL format to EndNote software for proper reference management.

### Study selection: inclusion and exclusion criteria (Step 3)

The third stage involved a rigorous two-phase screening process to identify eligible studies. In the first phase, two independent reviewers screened the titles, abstracts, and keywords of all unique records obtained after duplicate removal against the predefined inclusion and exclusion criteria (Table 2). Each reviewer performed their screening independently while blinded to the other reviewer’s decisions. Articles were excluded during this initial screening if they failed to meet criteria such as being in English, being clearly off-topic, not constituting peer-reviewed journal articles (e.g., books, theses, or reports), or having insufficient abstract/keyword information to assess eligibility.

**Table 2:** Inclusion and exclusion criteria.

Inclusion criteria	Exclusion criteria
Studies published in peer-reviewed journals from 2015–2024.	Studies that are older than 10 years
Studies exploring the link between global image and international war countries.	Studies that do not address the relationship between global image and countries involved in international war.
Studies that examine the global image of a country in international wars from different countries’ people (e.g. Western, Eastern, Middle Eastern, Asian, African, etc.) should be included.	Studies focused on purely domestic issues unrelated to international perceptions.
English-language publications.	Studies in non-English publications (unless translated)
Studies conducted following qualitative, quantitative as well as mixed methods.	Studies that do not have reliable and suitable methodology and lack quality and reliable data.

Following independent screening, both reviewers compared their results. Any discrepancies regarding inclusion or exclusion were flagged for resolution. These disagreements were addressed through discussion and consensus between the two primary reviewers. When consensus could not be reached, a third independent reviewer adjudicated the decision. This conflict resolution process ensured objectivity and minimized potential bias, resulting in 58 records advancing to full-text retrieval.

In the second phase, the full text of these 58 articles was retrieved and assessed using the same independent dual-reviewer process with conflict resolution as described above. Articles were excluded during full-text screening if detailed examination revealed they did not meet eligibility criteria, such as irrelevant focus, incorrect study type, or being unobtainable. This process yielded the final 56 articles included in the scoping review.

**Fig 1:**
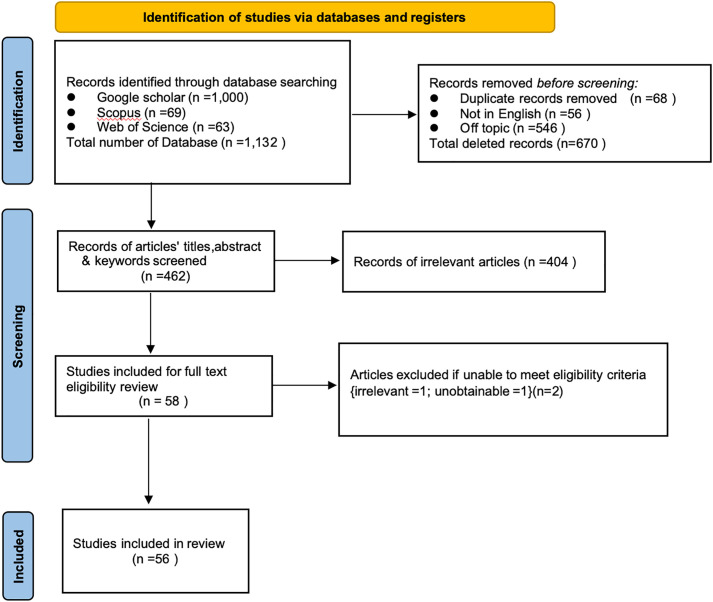
The PRISMA-ScR framework for the study selection procedure.

The detailed process and results of the screening, including the number of records excluded at each stage and reasons for exclusion, are presented in the PRISMA-ScR flow diagram in Fig 1. The narrower sample size likely results from our deliberate focus on war-related contexts. Furthermore, this can also be attributed to the nascent research stage on the global image of war, which is less studied than the national image.

### Data charting

We exported the retrieved articles, primarily used for content analysis, into an Excel spreadsheet Table 3. Before this, we identified the necessary details, including authors, source, instrument, research design, location, and influencing factors.

**Table 3:** Synthesis of selected articles.

Title	Authors	Source	Instrument	Research design	Author’s location	Influencing factors mentioned in the selected articles
A battle for foreign perceptions: Ukraine’s country image in the 2022 war with Russia	[26]	Scopus	Sentiment analysis	Qualitative	USA	Military and Historical
Armed violence in the system of state image formation	[27]	Google Scholar	Dialectical, systematic, structural-functional, institutional, conflict logical, political-cultural, and axiological approaches	Qualitative	Ukraine	Military and Geopolitical
Bringing War Back in: Victory and State Formation in Latin America	[28]	Google Scholar	Synthetic Control Method	Mixed,	UK	Military
Capturing Hearts: The Coverage of Iran’s Charm Offensive during the 2015 Nuclear Deal Negotiations in the American and Israeli Press	[29]	Google Scholar	Content analysis	Mixed	USA	Leadership
Challenges of branding in post-conflict countries: The case of Bosnia and Herzegovina	[30]	Google Scholar	Population survey and focus groups	Mixed	Croatia	Diplomatic
China’s image in U.S propaganda during the Pacific War era	[31]	Google Scholar	Document analysis	Qualitative	China	Diplomatic and Governmental
Cultural Diplomacy and Nation Branding in Modern Competitive International Environment	[32]	Google Scholar	Document analysis	Qualitative	Greece	Cultural and Diplomatic
Das Deutschlandbild: National Image, Reputation and Interests in Post-War Germany	[33]	Web of science	Historical analysis	Qualitative	Australia	Historical, Cultural, and Diplomatic
Democratic Reputations in Crises and War	[34]	Web of Science	Survey experiment	Quantitative	USA	Governmental and Military
Differentiated visibilities: RT Arabic’s narration of Russia’s role in the Syrian war	[35]	Google Scholar	Content analysis	Qualitative	UK	Diplomatic
Digital images and globalized conflict	[36]	Google Scholar	Content analysis	Qualitative	Denmark	Digital technology
Discriminant analysis of nation brands 2022 in terms of military invasion of Russian federation in Ukraine	[37]	Google Scholar	Discriminant analysis	Quantitative	Ukraine	Diplomatic and Cultural
Discursive battlefields: Support(ing) the troops in Canada	[38]	Google Scholar	Narrative analysis and discourse analysis	Qualitative	Canada	Leadership
Does using ‘imagefare’ as a state’s strategy in asymmetric conflicts improve its foreign media coverage? The case of Israel	[39]	Google Scholar	Narrative Analysis and Content Analysis	Mixed	Israel	Cultural and Diplomatic
Emotions and war on YouTube: affective investments in RT’s visual narratives of the conflict in Syria	[40]	Web of Science	Discourse analysis	Mixed	UK	Affective Investments and Cultural
Fighting over the image: the Israeli− Palestinian conflict in the Gaza strip 2018 − 19	[41]	Google Scholar	Narrative Analysis	Qualitative	Israel	Historical
Imagine there is war and it is tweeted live–An analysis of digital diplomacy in the Israeli-Palestinian Conflict	[42]	Google Scholar	Content analysis and expert interviews	Mixed	Germany	Governmental
Legitimation Discursive Strategies in President Biden’s Discourse about Russo-Ukrainian War	[43]	Google Scholar	Critical discourse analysis	Mixed	South Korea	Leadership
Mediated Public Diplomacy and Peace Journalism: International Public News Agencies on the Syrian Crisis	[44]	Google Scholar	Content analysis	Mixed	Turkey	Diplomatic
Military Conflicts and Country Image: The Country Image of Belligerents in Light of Ukraine, a Demographic, Communication Channel and Political Preference Based Perspective	[13]	Google Scholar	Questionnaire	Quantitative	Hungary	Governmental
Myth as Propaganda in World War I: American Volunteers, Victor Chapman, and French Journalism	[45]	Google Scholar	Historical documents	Qualitative	USA	Leadership and Historical
National Images as Integrated Schemas: Subliminal Primes of Image Attributes Shape Foreign Policy Preferences	[46]	Web of science	ANOVA and mediation analysis	Quantitative	USA	Subliminal Priming
One war, different coverage: Exploring cultural influences on international media framing of the Iraq War	[47]	Google Scholar	Content analysis	Qualitative	Nigeria	Cultural
Reassessing external images of the EU: Evolving narratives in times of crisis	[48]	Google Scholar	Interviews	Qualitative	Italy	Historical
Reimagining the Nation: Gendered Images of Italy and the Italo-Turkish War of 1911–12	[49]	Google Scholar	Historical analysis	Qualitative	USA	Historical and Leadership
Reputations and Change in International Relations	[50]	Google Scholar	Dynamic game-theoretic model	Quantitative	Turkey	Historical and Leadership
Reputations for Resolve and Higher-Order Beliefs in Crisis Bargaining	[51]	Google Scholar	Scenario-based survey experiment	Quantitative	UK	Historical and Military
Reputation of the Russian Federation After the Invasion of Ukraine	[52]	Google Scholar	Global Reputation Measurement	Quantitative	USA	Diplomatic
Research on the Image of China’s Anti-Japanese War on Major Global Social Media	[53]	Google Scholar	Content analysis	Qualitative	China	Historical
Revisiting Reputation: How Past Actions Matter in International Politics	[54]	Web of Science	Militarized Interstate Dispute	Quantitative	USA	Historical, Cultural and Leadership
Russian-Ukraine War: A comparative analysis of framing in Los Angeles Times and China Daily	[55]	Google Scholar	Discourse analysis	Qualitative	Netherlands	Diplomatic
Russia’s rising military and communication power: From Chechnya to Crimea	[56]	Google Scholar	Content analysis	Qualitative	UK	Leadership, Military and Diplomatic
Social media, Stereotypes, and the Acknowledgement of War Crimes	[57]	Google Scholar	Focus groups	Mixed	UK	Leadership
The Finest Feats of the War? The Captures of Baghdad and Jerusalem during the First World War and Public Opinion throughout the British Empire	[58]	Google Scholar	Content analysis	Qualitative	Canada	Military
The Gulf Crisis and Rise of Digital Nationalism in Qatar: A Case Study of Tweets	[59]	Google Scholar	Analysis of tweets and hashtags	Qualitative	Qatar	Diplomatic
The Image of Russia in the Western Press as a «Military Threat» Tool: Following the Media Content	[60]	Google Scholar	Linguistic discourse analysis, Cultural analysis, and Content analysis	Mixed	Russia	Governmental
The impact of anti-democratic actions on a country’s international image, as projected in the foreign press and on social media: The case of Israel	[61]	Google Scholar	Content analysis	Quantitative	Israel	Anti-democratic actions
The Impact of the Second Anglo-Boer War (1899–1902) on Spain’s Public Opinion, Press, Literature and International Image	[62]	Google Scholar	Historical analysis	Qualitative	Spain	Historical and Diplomatic
The Media Image of Ukraine: An European View	[63]	Google Scholar	Content analysis and discourse analysis	Mixed	Ukraine	Diplomatic and Geopolitical
The race for revision and recognition: Interwar Hungarian cultural diplomacy in context	[64]	Google Scholar	Historical analysis	Qualitative	Hungary	Diplomatic
The Reputational Cost of Military Aggression: Evidence from the 2022 Russian Invasion of Ukraine	[65]	Web of science	Pre-post survey design	Quantitative	USA	Military and Geopolitical
The return of the hero-leader? Volodymyr Zelensky’s international image and the global response to Russia’s invasion of Ukraine	[66]	Google Scholar	Content analysis	Qualitative	Poland	Leadership
The role of diplomatic services in shaping international image: Ukrainian diplomacy before and after 24 February 2022	[67]	Google Scholar	Case Study	Qualitative	Ukraine	Diplomatic
The Role of Direct Contact in Forming Peace-Oriented International Relations of a Divided Country: Focusing on the Division of the Korean Peninsula	[68]	Web of science	Self-questionnaires	Quantitative	South Korea	Historical
The Ukraine War as an Exogenous Shock for the Image of Russia	[69]	Google Scholar	Descriptive trends and statistical modeling	Mixed	USA	Historical and Leadership
To Whom the Sirens Wail.” Poland’s Post-2022 Geopolitical Debates on Central and “Eastern Europe	[70]	Google Scholar	Discourse analysis	Qualitative	Czech	Historical and Geopolitical
Toward a History of Interwar Sino-Hungarian Cultural Relations: Three Advocates of Kuomintang Soft Power, Hungarian Irredentism and Pan-Danubianism	[71]	Google Scholar	Historical analysis	Qualitative	USA	Leadership
Transiting From the East to the ‘Core’ West of Europe: Slovakia’s Ontological Liminality After the Outbreak of 2022 Russia’s War on Ukraine	[72]	Google Scholar	Discourse Analysis	Qualitative	Slovakia	Geopolitical, Diplomatic and Historical
Ukraine in the mirror of foreign online media after the beginning of the war in Israel	[73]	Google Scholar	Content analysis and. Discourse analysis	Quantitative	Ukraine	Leadership
UKRAINIAN WARTIME NATION BRANDING DURING THE RUSSO-UKRAINIAN WAR	[74]	Google Scholar	Critical discourse analysis	Qualitative	Ukraine	Historical and Leadership
Using social media in the news reportage of War and Conflict: Opportunities and Challenges	[75]	Google Scholar	Semi-structured interviews	Qualitative	Switzerland	Diversity of information sources
Visual images as affective anchors: strategic narratives in Russia’s Channel One coverage of the Syrian and Ukrainian conflicts	[76]	Google Scholar	Content analysis	Mixed	Finland	Historical, Cultural and Visual Tropes
Visually framing the Gaza War of 2014: The Israel Ministry of Foreign Affairs on Twitter	[77]	Google Scholar	Content analysis	Mixed	UK	Governmental
War on frames: Text mining of conflict in Russian and Ukrainian news agency coverage on Telegram during the Russian invasion of Ukraine in 2022	[78]	Google Scholar	Text Mining	Mixed	Poland	Geopolitical
War on Terror Impacts on Social Fabric: Intra-State Sectarianism and Social Cohesion in Pakistan	[79]	Google Scholar	Secondary data analysis	Qualitative	Pakistan	Military
When the Media Goes to War: How Russian News Media Defend the Country’s Image During the Conflict with Ukraine	[80]	Google Scholar	Thematic content analysis	Qualitative	USA	Governmental

Data charting followed a structured dual-reviewer protocol to ensure consistency. After developing an extraction template (Table 3) and factor categorization codebook, two trained reviewers independently charted all studies. The process included pilot calibration, inter-rater reliability testing (κ = 0.83) (Cohen’s Kappa), and consensus-based conflict resolution. Influencing factors were identified verbatim from source texts, categorized using our hybrid deductive-inductive framework, and validated through dual verification. This rigorous process underpins the factor frequencies reported in Table 4.

**Table 4:** Frequency of influencing factors (in descending order) on the global image of war-involved countries, 2015-2024.

Influencing factors	Frequency	2024	2023	2022	2021	2020	2019	2018	2017	2016	2015
Historical	17	4	6	/	3	/	/	2	/	/	2
Diplomatic	16	3	2	3	4	/	1	2	/	1	/
Leadership	14	3	5	1	/	1	/	1	1	/	2
Military	9	1	4	1	2	/	/	/	1	/	/
Governmental	7	2	1	/	1	/	1	1	1	/	/
Cultural	7	/	/	2	2	1	/	1	/	1	/
Geopolitical	6	3	1	1	1	/	/	/	/	/	/
Digital technology	1	/	/	/	/	/	/	/	1	/	/
Affective Investments	1	/	/	/	/	1	/	/	/	/	/
Subliminal Priming	1	/	/	/	/	/	/	/	/	1	/
Diversity of information sources	1	/	/	/	/	/	/	/	/	/	1
Visual Tropes	1	/	/	/	1	/	/	/	/	/	/
Total	81	18	19	8	14	3	2	7	4	3	5

### Results

**Fig 2:**
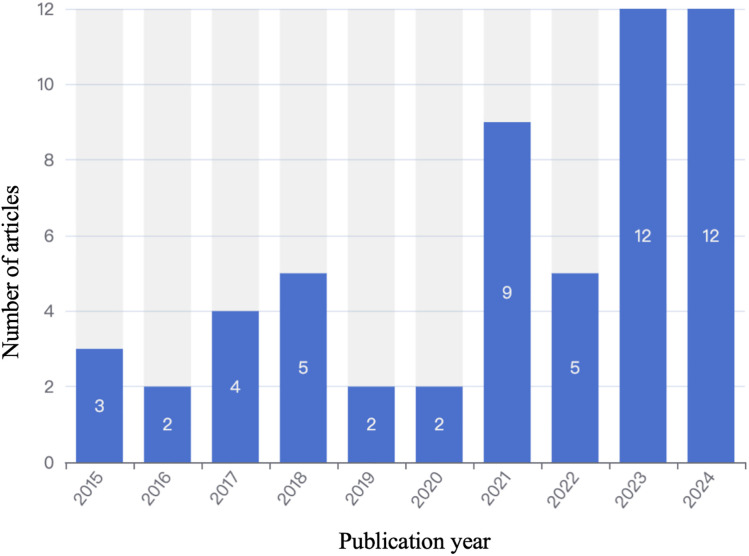
The publication trends of papers on the global image in war from 2015 to 2024.

As shown in Fig 2, our analysis of publication patterns reveals a notable fluctuation in academic interest over the past decade, with a clear surge corresponding to major international conflicts/wars. From 2015 to 2020, annual publications remained relatively low, ranging from 2 to 5 articles per year. A significant increase was observed in 2021, with 9 articles published, followed by a slight dip to 5 articles in 2022. However, a sharp rise occurred in 2023, with 12 articles, and this level was sustained through 2024, also with 12 articles. This trend aligns closely with the timing of the Russo-Ukrainian War (2022) and other regional escalations, highlighting how contemporary wars stimulate scholarly focus on image dynamics.

**Fig 3:**
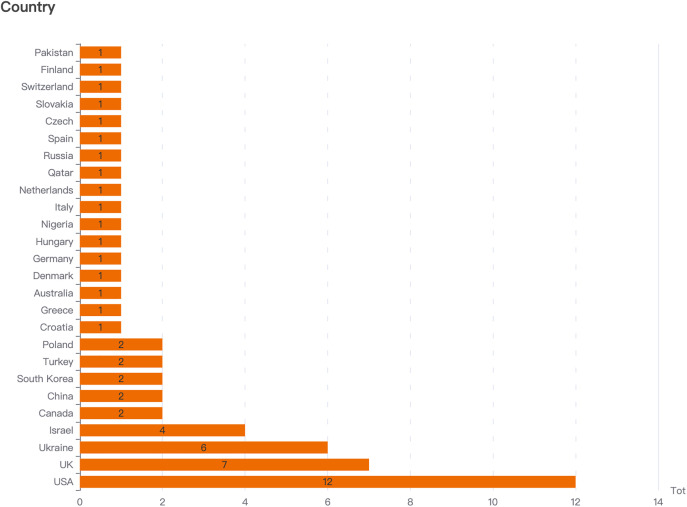
Countries and number of papers published on factors influencing global image from 2015 to 2024.

Geographically (Fig 3), research output is concentrated in countries with significant geopolitical influence or direct conflict/war involvement. The United States (12 articles), the United Kingdom (7 articles), and Ukraine (6 articles) dominate, reflecting their central roles in global affairs and academia. Israel (4 articles) is also prominently represented. Additionally, Canada, China, South Korea, Turkey, and Poland each contributed 2 articles. A further 17 countries—including Pakistan, Finland, Switzerland, Slovakia, the Czech Republic, Spain, Russia, Qatar, the Netherlands, Italy, Nigeria, Hungary, Germany, Denmark, Australia, Greece, and Croatia—each contributed 1 article. This distribution indicates both a concentration of research in Western and conflict-adjacent nations, while also revealing a diverse, albeit sparse, global engagement with the topic.

Table 4 shows the analysis of the extracted data. In the included studies, there was a clear pattern in the frequency with which different factors were considered to influence global image during war. Historical factors (n = 17), Diplomatic factors (n = 16), and Leadership factors (n = 14) emerged as the most frequently cited and prominent categories. Consequently, these three factors constitute the core focus of our detailed thematic synthesis below, as they represent the most substantial and recurrent themes within the current literature landscape captured by this review. The less frequently cited factors are categorized as secondary factors (e.g., Military (n = 9), Governmental (n = 7), Cultural (n = 7), Geopolitical (n = 6)) and emerging factors (e.g., Digital Technology, Affective Investments, Subliminal Priming, Diversity of information sources, Visual Tropes, n = 1 for each), which are synthesized collectively in “Additional Contributing Factors to Wartime Global Image”.

### Core factors

#### Historical factors (n=17).

The included studies identify historical factors primarily as past wars, conflicts, colonial legacies, unresolved historical issues, collective memory, and state-led narrative framing of these histories (e.g., [53,62,69,70]). Our synthesis reveals that historical factors as foundational yet contested elements shaping wartime global image through four interconnected mechanisms. Synthesis across 17 studies demonstrates these mechanisms operate within specific contextual constraints, generating both enduring legacies and strategic opportunities:

(1) **Providing Cognitive Frameworks.** Historical events supply potent analogies and reference points that structure global media and public interpretation of current conflicts. These frameworks actively shape perceptual lenses, making certain interpretations more salient. For instance: China’s reconstruction of Anti-Japanese War imagery provided a ready-made framework for understanding contemporary geopolitical stances [53]. Comparisons of Russian actions in Ukraine to Nazi aggression leveraged World War II symbolism to frame current events within a widely understood narrative of unprovoked aggression [26]. The effectiveness of historical analogies depends on their recognizability and perceived relevance to the international audience [70]. Overly reductive or contested analogies can backfire or polarize.
(2) **Triggering Affective & Moral Responses.** Specific historical traumas or triumphs evoke powerful emotions (sympathy, fear, resentment, admiration) that anchor moral evaluations of conflict actors. These responses often bypass rational deliberation. References to Spain’s colonial losses or the suffering during the Spanish Civil War continue to evoke national pathos, impacting external perceptions [62]. Ukraine’s mobilization of narratives centered on historical resistance (e.g., Cossack heritage, Holodomor) fostered international sympathy by framing the current conflict as another chapter in a long struggle for survival [69]. The emotional potency of history is amplified in conflicts with deep historical roots (e.g., Israel-Palestine [41]), where contemporary violence reactivates collective trauma.
(3) **Enabling Intent & Credibility Assessments.** Past behavioral patterns become heuristics for judging a state’s current reliability, intentions, and likely adherence to norms during war. Research on reputations (e.g., for resolve or treaty adherence [50,51,54]) shows how past actions influence calculations of alliance trustworthiness and deterrence efficacy. A state perceived as historically unreliable faces greater skepticism. Germany’s post-WWII commitment to pacifism and multilateralism, built over decades, initially shaped expectations (and criticisms) of its response to the Ukraine invasion [33].
(4) **Serving Legitimization/Delegitimization.** Historical narratives are actively weaponized by warring parties to justify their own actions and discredit adversaries. States deliberately invoke or reconstruct history: Ukraine framed its resistance as defending “European values” against a recurring Russian imperial threat [26], while Russia portrayed its actions as correcting historical injustices or resisting NATO encroachment (framed as a continuation of Western hostility) [69,72,76]. Embedding adversaries within negative historical analogies (e.g., Nazi comparisons [26], colonial oppressors) is a common delegitimization tactic [45,53]. This is a contested terrain. Multiple actors (states, media, diaspora, civil society) actively reinterpret the past. Spain’s strategic “taming” of Civil War memory demonstrates state efforts to manage sensitive history for contemporary image goals [62].

We conclude that historical factors function as a dynamic interpretive battleground rather than a static backdrop. Their influence stems from the interplay between deeply ingrained collective memories/emotions and the strategic agency of actors who selectively mobilize, reframe, or suppress aspects of the past. While historical legacies create path dependencies shaping perceptions of intent and moral standing, their impact is contingent on the strategic skill with which narratives are constructed and disseminated, their resonance with pre-existing international understandings, and the specific historical depth of the conflict context. History’s potency lies in its dual role: providing seemingly objective frameworks for understanding present chaos while simultaneously serving as a highly subjective tool for wartime justification and condemnation. Successfully navigating this terrain requires states to acknowledge the weight of history while strategically managing its narrative to align with contemporary image goals, mindful of the risks of oversimplification and the contested nature of historical memory itself.

#### Diplomatic factors (n=16).

Diplomatic Factors—encompassing strategic communication, alliance building, public diplomacy, and crisis management—prove instrumental in shaping wartime global images. States actively deploy narrative framing to dominate conflict interpretations, exemplified by Qatar’s leveraging of Al Jazeera during the Gulf crisis [59]. Concurrently, alliance signaling serves as a legitimacy anchor, with Ukraine’s NATO aspirations [63] and Qatar’s trilateral cooperation with Western powers [59] demonstrating how multilateral endorsements amplify perceived credibility. Soft power initiatives further buffer negative perceptions: Germany’s cultural diplomacy is a key tool for dispelling historical stigma [33], while Turkey’s humanitarian response to Syrian refugees reframed its regional role [44].

Our critical analysis reveals four key tensions: **(1) Alliance Building and Signaling as Legitimacy Anchors.** Forming, maintaining, and signaling alliances is a critical diplomatic tool for wartime image management. Multilateral endorsements and cooperation serve as powerful signals of legitimacy and credibility. Ukraine’s pursuit of NATO membership and Western support became central to its image as a victim defending democratic values against aggression, garnering significant international sympathy and aid [63]. Qatar’s trilateral cooperation with the US, France, and Turkey during the Gulf Crisis provided tangible diplomatic backing that bolstered its global image and resilience [59]. Conversely, Russia’s diplomatic isolation following its invasion of Ukraine served to severely damage its international reputation [52].

(2) **Soft Power and Humanitarian Diplomacy as Buffers.** Diplomatic efforts extend beyond immediate conflict justification to include soft power initiatives and humanitarian actions that can mitigate negative perceptions or reframe a country’s role. Turkey’s humanitarian response to Syrian refugees, framed through diplomatic channels and international news agencies, played a role in reshaping its regional image during the Syrian crisis [44]. Germany’s sustained post-war cultural diplomacy has been instrumental in dispelling historical stigma and rebuilding trust, demonstrating the long-term image value of soft power [33]. However, Bosnia and Herzegovina’s experience highlights the limitations of soft power/branding efforts when underlying political and ethnic tensions remain unresolved, hindering effective post-conflict image rehabilitation [30].
(3) **Crisis Communication and Responsiveness.** The ability to manage diplomatic communications swiftly and effectively during rapidly evolving crises is paramount. This involves addressing accusations, providing updates, and managing international expectations. Studies on real-time digital diplomacy (e.g., Ukraine, Israel-Palestine) emphasize the importance of agility and responsiveness in this domain [39,50,52,63].
(4) **Strategic Narrative Framing and Information Dominance.** States actively deploy diplomatic communication to construct and disseminate narratives framing the conflict, their role, and their adversary’s actions. This aims to dominate the international interpretation of events. For instance: Qatar leveraged Al Jazeera during the Gulf Crisis to project its narrative and counter regional rivals, significantly influencing its international standing [59]. Israel’s “digital hasbara” strategy utilizes social media for real-time rebuttals and narrative control in asymmetric conflicts [39,50]. Russia employed sophisticated communication strategies, often through state-aligned media like RT Arabic, to justify its actions in Syria and Ukraine, attempting to shape perceptions particularly in specific regional audiences [35,56].

We argue that diplomatic efforts function as a complex ecosystem of influence where narrative construction, alliance politics, credibility management, and soft power leverage interact dynamically to shape wartime perceptions. Success is fundamentally contingent on the strategic alignment of rhetoric, action, and identity: narratives must resonate authentically with target audiences and be substantiated by observable state behavior and policy choices. While digital tools offer unprecedented reach and speed, they amplify the risks of the credibility-audience paradox. Crucially, diplomacy’s efficacy is not uniform; smaller states or those under sanctions can exploit niche strategies or counter-narratives, but all actors operate within the constraining force of international scrutiny. Effective wartime diplomacy thus requires navigating a tension between immediate narrative control and the long-term cultivation of credibility through consistent, values-aligned actions that reinforce the state’s projected identity on the global stage.

#### Leadership factors (n=14).

Our analysis of the 14 articles reveals leadership as a dynamic and multifaceted factor shaping wartime global image through three primary mechanisms: symbolic representation, strategic communication, and crisis decision-making. Synthesis across studies indicates these mechanisms operate within specific contextual constraints and generate distinct perceptual outcomes. Its symbolic representation is that of a leader who embodies the Nation.

(1) **Symbolic representation.** Studies converge on leaders becoming potent symbolic anchors during conflict, personifying national resolve, values, and victimhood/perpetrator status. Volodymyr Zelensky’s transformation into a global symbol of democratic resistance exemplifies this [66,73,74]. His ubiquitous media presence, military attire, and direct appeals fostered international identification with Ukraine as the “underdog.” Conversely, leadership symbolism can also reinforce negative images. Analysis of Russian leadership communication framed actions within narratives of historical grievance and national restoration, which resonated domestically but solidified perceptions of aggression abroad [56].
(2) **strategic communication.** Strategic communication mainly manifests itself in Controlling the Narrative. Leadership directly shapes image through deliberate media engagement and message control. Studies document leaders and elites using platforms (traditional media, social media) to frame conflicts, justify actions, demonize opponents, and appeal for support (“digital hasbara,” strategic wartime tweets) [29,73,74]. President Biden’s discursive strategies regarding the Russo-Ukrainian War, employing legitimization frames (e.g., democracy vs. autocracy), were identified as instrumental in rallying Western support and shaping perceptions of US leadership [43]. The credibility of leadership communication is paramount. Studies highlight a tension: while real-time, direct communication (e.g., Zelensky’s social media) can build empathy and bypass traditional media gatekeepers [73], overly controlled propaganda or messages contradicted by evidence can backfire, damaging international trust [29,43,50,73].
(3) **Crisis decision-making.** Crisis Decision-Making and Action is Demonstrating Resolve and Values. Leaders’ concrete actions during war significantly impact the image. Decisions regarding military strategy, humanitarian conduct, alliance management, and adherence to international norms are scrutinized globally. Perceived resolve and competence are critical dimensions. Research on reputational costs suggests that leaders perceived as vacillating or ineffective can damage their nation’s image and embolden adversaries [50]. Conversely, decisive actions aligned with stated values (e.g., organizing refugee aid [71]) bolster positive perceptions. Leadership transitions introduce significant uncertainty. Leadership changes can disrupt established reputations for resolve or diplomatic postures, impacting how allies and adversaries perceive the nation’s commitment and predictability [50].

We conclude that leadership is not a monolithic factor but operates through interconnected symbolic, communicative, and behavioral channels. Its impact on global image is highly contingent on the perceived consistency between rhetoric and action, the leader’s ability to project authenticity and resolve that resonates with target audiences, and the specific context of the conflict (e.g., perceived legitimacy of the cause, democratic vs. authoritarian setting). While charismatic leadership can provide a powerful national image boost (e.g., Zelensky), it cannot fully offset the reputational damage caused by actions widely condemned by the international community.

### Secondary factors

#### Military factors (n=9).

Military factors encompass a state’s actual military conduct, strategic execution, and its direct impact on its global image. Our comprehensive analysis reveals that military factors significantly shape a country’s image through the following mechanisms.

(1) **Operational Effectiveness and Tactical Execution.** Military successes or failures directly signal national strength and resolve. For instance, Ukraine’s successful defense in the early stages of the 2022 war (e.g., the Battle of Kyiv) rapidly enhanced its image as a “resilient underdog” [26]. Conversely, military setbacks or retreats may be interpreted as weakness or strategic failure, as seen in Russia’s struggles in certain Ukrainian campaigns, which damaged its reputation as a military power [65].
(2) **Compliance with International Humanitarian Law and Conduct.** Adherence to the laws of war (e.g., distinguishing military from civilian targets, treating prisoners of war) serves as a key basis for moral judgment. Violations of international law (e.g., attacks on civilian infrastructure) trigger strong international condemnation and reputational damage [65], while restrained and professional military behavior may garner respect or even sympathy.

Military factors do not operate in isolation, their impact on the image highly depends on diplomatic narratives (e.g., whether military actions are successfully justified) and leadership symbolism (e.g., whether leaders personally visit frontline troops). Moreover, the visual representation of military actions (e.g., drone footage, war correspondent reports) amplifies these effects through digital platforms, further strengthening or undermining image projection [36].

#### Cultural factors (n=7).

Cultural factors indirectly shape how the international community interprets a country’s wartime actions through soft power, cultural affinity, and value-based communication.

(1) **Cultural Affinity and Narrative Resonance.** Culturally similar countries or regions more easily understand and accept certain narratives. For example, Western media coverage of the Iraq War was significantly influenced by cultural values—U.S. media emphasized a “liberation” narrative, while Arab media focused more on civilian casualties and resistance [47].
(2) **Cultural Diplomacy and Nation Branding.** Long-term cultural outreach (e.g., language promotion, artistic exchanges, educational cooperation) helps build a “reservoir of credibility” that can mitigate negative perceptions during crises. Germany, through sustained cultural exchange and historical reflection, successfully reshaped its post-war image, allowing it to act as a “mediator” rather than an “aggressor” during the Ukraine crisis [33].

The influence of cultural factors tends to be delayed and long-term. While their effect may be less immediate than diplomatic or military factors during intense warfare, they play a crucial role in post-conflict reconstruction and long-term image rehabilitation.

#### Governmental factors (n=7).

Governmental factors relate to a state’s political system, governance efficacy, and domestic political coherence, providing the background against which the international community assesses its credibility and legitimacy.

(1) **Political System and Democratic Credibility.** Democratic countries are generally perceived as more transparent and accountable, making their wartime actions more likely to gain international trust. Research indicates that democratic states more easily maintain their reputations during crises [34]. In contrast, authoritarian regimes often face skepticism regarding their intentions, as seen in Russia’s portrayal in Western media as an “unpredictable aggressor” [60].
(2) **Domestic Political Coherence and Stability.** Internal political divisions or policy inconsistency can undermine international confidence in a country’s wartime commitments. For example, delays in U.S. aid to Ukraine amid bipartisan disputes were interpreted as “commitment uncertainty”, affecting its image as a reliable ally [34].

Governmental factors provide “background credibility” for diplomatic and military behavior. Their influence, though indirect, is profound—especially in prolonged conflicts and post-war phases.

#### Geopolitical factors (n=6).

Geopolitical factors involve a state’s position in the international system, its alliance relationships, and strategic interests, which precondition how the international community interprets its actions.

(1) **Alliance and Bloc Affiliation.** A country’s membership in alliance systems (e.g., NATO, CSTO, Non-Aligned Movement) directly influences its image positioning. Ukraine’s status as a “NATO candidate” naturally garnered sympathy and support from Western blocs [63], while Russia’s antagonistic geopolitical stance toward the West placed it within an “adversarial” narrative [65,70,72,78].
(2) **Historical Geopolitical Rivalries and Memory.** Historical geopolitical rivalries (e.g., Russo-Ukrainian relations, Middle Eastern conflicts) persistently influence contemporary image judgments. For example, Poland’s historical fear of Russia made it one of the most vocal critics of Moscow during the Ukraine war [70].

Geopolitical factors essentially act as “structural filters”, determining which historical narratives are activated, which diplomatic signals are trusted, and which leadership behaviors are amplified or ignored.

### Emerging factors

Beyond the core and secondary factors previously discussed, our review identified several emerging and niche factors that warrant attention. While the body of literature for each is currently smaller, they represent important and evolving dimensions of how wartime images are formed. Such as the role of digital technology in information dissemination [36], affective or emotional dimensions of media consumption [40], diversity of Information Sources used by audiences or media [75], and subliminal priming effects [46]. Their influence is often amplified by and interwoven with the core factors, operating as force multipliers or disruptive elements within the modern information ecosystem.

#### Digital Technology (n=1).

Digital technology fundamentally reconfigures the very battlefield of image formation. It is not merely a communication channel but an environment that shapes how narratives are constructed, disseminated, and consumed in near real-time. Blaagaard et al. [36]highlight how digital images contribute to globalized conflict perception, creating immediate and visceral connections to distant wars. This factor underpins the modern context in which all other factors (e.g., diplomatic communication, leadership symbolism) are amplified and contested.

#### Affective Investments (n=1).

This factor moves beyond rational or ideological persuasion to focus on the deliberate cultivation of emotional responses to shape moral alignment and audience allegiance. Crilley & Chatterje-Doody [40], demonstrate how Russia’s RT used visual narratives of the Syria conflict to make deliberate affective investments, eliciting emotions like sympathy (for civilians) or outrage (against perceived aggressors) that shape audience alignment and moral evaluation of conflict parties beyond rational analysis.

#### Subliminal Priming (n=1).

This factor operates at a subconscious level, suggesting that image formation is influenced by cues that audiences are not actively aware of, revealing a psychological depth to image perception. Castano et al. [46] provide experimental evidence that subliminal primes of national image attributes (e.g., “modern” vs. “traditional”) can directly shape individuals’ foreign policy preferences. This indicates that a country’s image operates as a cognitive schema—a network of associated attributes—that can be activated automatically. Unlike overt propaganda, subliminal priming works by subtly activating these schemas, thereby influencing how subsequent information about the country is interpreted. This makes it a powerful tool for reinforcing existing perceptions or gently nudging attitudes without triggering counter-arguing. This mechanism suggests that the effectiveness of diplomatic messages, leadership symbolism, or historical analogies may be partly dependent on the pre-existing, often subconsciously held, image schemas they activate in the target audience.

#### Diversity of Information Sources (n=1).

The fragmentation of the media landscape challenges state-centric image management and creates a more complex environment for narrative control. Sacco & Bossio [75] discuss how the use of social media in war reportage diversifies the information ecosystem. Citizens, soldiers, and NGOs become direct sources of information, creating a polyvocal environment that can challenge official state narratives (e.g., user-generated content contradicting claims of military precision). This diversity presents both an opportunity and a challenge. While it allows for a multiplicity of perspectives, it also creates a saturated environment where misinformation thrives and the veracity of information becomes a central battleground, impacting the credibility of all actors. This factor highlights the increased agency of audiences in curating their own information diets, meaning states must compete for attention and credibility within a much wider and more decentralized field of sources.

#### Visual Tropes (n=1).

The strategic use of recurring visual themes is a key mechanism for simplifying complex conflicts and solidifying narratives through memorable and emotionally charged imagery. Grigor & Pantti [76] analyze how Russia’s Channel One employed specific visual tropes—such as images of humanitarian aid delivery (shaping Russia as a helper) or the spectacle of military precision (shaping it as strong)—as “affective anchors” for its strategic narratives on Syria and Ukraine. Visual tropes are highly effective because they are easily processed, remembered, and shared. They reduce complexity to a single, powerful frame (e.g., the “heroic defender,” the “brutal aggressor,” the “innocent victim”). The power of visual tropes is immensely amplified by digital platforms, which facilitate their rapid viral spread. A single powerful image or video clip can become an iconic representation of the entire conflict, defining a country’s image for a global audience.

These emerging factors collectively depict a shift in the ontology of wartime image formation. The process is no longer dominated solely by slow-moving, state-driven diplomacy and traditional media. Instead, image is increasingly formed in a dynamic, digital, and affective battleground characterized by: (1) Narratives can be launched and challenged instantaneously (Speed and Volatility). (2) States compete with non-state actors and even individuals for narrative control (Multi-Actor Participation). (3) Appeals to emotion are as important as appeals to reason or fact (Emotional Resonance). (4) Image cues operate both consciously and subconsciously (Psychological Depth). (5) The visual register is a primary site of contestation (Visual Primacy).

While less frequently emphasized than Historical, Diplomatic, and Leadership factors within the reviewed literature, these additional elements contribute to a more nuanced understanding of the multifaceted forces shaping global image during conflict. Their identification is critical for a comprehensive scoping review, as they point to evolving mechanisms of image formation—particularly digital, affective, and psychological—that are likely to grow in importance. Future research should focus on expanding the empirical investigation of these factors.

## Discussion

In this review, we systematically mapped 56 studies (2015–2024) for identifying factors shaping a country’s global image during international wars are systematically mapped. Our synthesis reveals that historical legacies, diplomatic strategies, and leadership actions constitute the most prominent and recurrent influences, operating as interconnected pillars rather than isolated forces. They form an interdependent ecosystem, the efficacy of which is critically mediated and moderated by a suite of secondary and emerging factors. Below, we critically analyze this integrated system, its mechanisms, interactions, and tensions, and propose targeted future research.

We argue that historical factors as contested battlegrounds. History functions not merely as background but as an active interpretive framework leveraged by states. Its power lies in four mechanisms: (1) supplying cognitive analogies (e.g., WWII comparisons framing Russia as aggressor [26]); (2) triggering affective responses (e.g., sympathy for Ukraine’s “victim” narrative [69]); (3) enabling credibility assessments (e.g., Germany’s pacifist reputation shaping expectations [33]); and (4) serving legitimization tools (e.g., Russia’s “anti-NATO” rhetoric [76]). Crucially, this is a strategic battlefield: success depends on narrative resonance with global audiences and alignment with observable actions. Overly reductive analogies (e.g., ubiquitous “Nazi” comparisons) risk polarization or backfiring [70].

Diplomatic efforts, such as narrative framing, alliance signaling, and crisis communication, succeed only when rhetoric aligns with behavior. While Qatar skillfully deployed Al Jazeera to project legitimacy during the Gulf Crisis [59], and Ukraine’s NATO aspirations amplified its democratic victimhood [63], ineffective diplomacy exacerbates negative perceptions (e.g., Russia’s post-invasion isolation [52]). We identify a double-edged dynamic: digital tools enable real-time outreach (e.g., Zelensky’s social media [73]), but accelerate scrutiny of inconsistencies. Smaller states (e.g., Qatar) can exploit niche strategies, yet all actors face intensified audience costs when actions contradict diplomatic claims [39,44].

Leaders personify national resolve, but their impact hinges on perceived authenticity. Zelensky’s transformation into a global “resistance icon” [73,74] demonstrates how symbolic representation and direct communication can reshape narratives. However, leadership is brittle: perceived hypocrisy (e.g., Iran’s “charm offensive” undermined by hardline policies [29]) or vacillation damages credibility. Leadership transitions also disrupt reputational continuity (e.g., shifts in U.S. foreign policy posture [50]). Charisma may buffer image damage temporarily, but cannot offset egregious violations of international norms [61].

Three core factors function as a dynamic, interdependent system. Historical factors provide the foundational narratives and affective lenses (e.g., WWII analogies framing the Russia-Ukraine war [26,69]), but their potency is not automatic. Their impact is contingent on diplomatic skill in framing and disseminating them [59,63] and on leadership’s authenticity in embodying them [73,74]. Conversely, diplomatic efforts, such as narrative framing and alliance signaling, succeed only when their rhetoric is congruent with a state’s historical reputation [33] and is credibly enacted by its leadership [43]. Leadership, in turn, derives its symbolic power from its alignment with resonant historical narratives [74] and its ability to leverage diplomatic channels effectively [39]. This triad forms the primary engine of image formation, but its output is fundamentally filtered through other critical factors.

The influence of the core triad is not deterministic, does not operate in a vacuum, but forms a dynamic, interdependent system continuously shaped by secondary and emerging factors. Rather than functioning in isolation, factors such as military conduct, cultural proximity, and digital technology interact with and are mediated by the historical, diplomatic, and leadership pillars.

Military and Governmental Factors as Action-Credibility Links. The military factor constitutes the most direct and visceral manifestation of state action. However, its impact on the image is not merely a function of battlefield success but is interpreted through the core factors. A militarily successful action (e.g., the defense of Kyiv) can be dramatically amplified into a powerful image of resilience if it is successfully framed within a historical narrative of David vs. Goliath [26,69] and championed by authentic leadership communication [73,74]. Conversely, military actions perceived as violations of international law can cause severe reputational damage [65], but the extent of this damage is often mitigated or exacerbated by diplomatic crisis management [52] and the credibility of governmental institutions [34]. Governmental factors thus provide the foundational credibility (or lack thereof) against which diplomatic rhetoric and leadership claims are judged. Anti-democratic actions [61] or perceived internal instability erode this foundation, making subsequent diplomatic and leadership narratives less credible internationally.

Cultural and Geopolitical Factors as Receptivity Lenses. Cultural factors influence how diplomatic narratives resonate with different international audiences. The same diplomatic message may be received differently in culturally proximate versus distant regions, as evidenced by studies on the varied media framing of wars (The Iraq War) [47]. This means a diplomatic narrative effective in one cultural context may fail or backfire in another. Similarly, geopolitical factors create pre-existing alignments and rivalries that predispose audiences to interpret a nation’s actions through a lens of strategic interest or historical allegiance [63]. A state’s geopolitical position acts as a powerful filter, determining which historical analogies resonate and which diplomatic alliances are perceived as legitimate.

Digital, Affective, and Cognitive Factors as the Modern Battlespace. The digital technology factor fundamentally transforms the arena in which leadership and diplomacy operate. It amplifies leaders’ ability to communicate directly and symbolically (e.g., Zelensky’s social media posts [73]) but also accelerates the global scrutiny of inconsistencies between a state’s diplomatic rhetoric and its actions on the ground [39,63]. Within this digital arena, affective investments [40] and visual tropes [76] are the primary weapons for triggering the emotional responses (e.g., sympathy, outrage) that underpin historical narratives. They allow states to create immediate, visceral connections to distant conflicts, bypassing rational analysis. Furthermore, the diversity of information sources [75] fragments the media landscape, challenging state attempts at narrative control and creating a constant struggle for visibility and credibility. At a subtler level, psychological level, subliminal priming [46] research suggests that deeply held historical image schemas can be activated to influence foreign policy preferences almost unconsciously, pointing to a profound psychological layer of image formation.

In essence, we argue that while historical, diplomatic, and leadership factors provide the primary frameworks for image formation, the secondary and emerging factors act as critical amplifiers, filters, and moderators that determine the ultimate potency and reception of a nation’s projected image during war.

## Conclusion

This review concludes that a country’s global image during war is not built on a single pillar but forged in a dynamic arena. The core triad (History, Diplomacy, Leadership) generates the primary narrative content. However, the ultimate success of this narrative is determined by how it is credibilized by military and governmental actions, filtered through cultural and geopolitical lenses, and contested on the digital-affective-cognitive battleground. This integrated model moves beyond simplistic, media-centric or state-centric explanations to offer a more nuanced, multi-level framework for understanding the complex process of global image formation in times of war. It illustrates that winning the image war requires not only controlling the narrative but also understanding the perceptual terrain upon which it is received. Isolated tactics will likely fail against the global scrutiny of factor coherence.

### Limitations of the scoping review

We acknowledge that this review is subject to several methodological and conceptual constraints that should be considered when interpreting its findings.

### Time-based constraints

Our exclusive focus on literature from 2015–2024 may overlook seminal works, limiting insights into long-term evolutionary trends in wartime image formation.

### Methodological limitations

Our search strategy was limited to three major databases (Web of Science, Scopus, and Google Scholar) and relied predominantly on the term ‘image’ as the core keyword. Although preliminary searches indicated that alternative terms like ‘perception’ and ‘reputation’ did not yield substantially different results, it is possible that some relevant studies employing different terminology were inadvertently missed. Furthermore, we also recognize that the restriction to English-language, peer-reviewed journal articles may have introduced language and publication biases, potentially marginalizing valuable perspectives published in other languages or in non-journal formats (e.g., books, reports).

### geographical and conceptual skew

Geographically, the overrepresentation of studies from the U.S., U.K., Ukraine, and Israel (Fig 3) underscores a Western-centric skew in the extant literature. This may obscure unique factors that shape the global image of nations in the Global South during conflicts. Thematically, while the dominance of historical, diplomatic, and leadership factors justified their prioritization, this emphasis may have inadvertently underrepresented emerging influences such as digital technology affordances, affective investments, or subliminal priming, which were identified in fewer studies.

### Contextual boundaries

Conceptually, our state-centric framework excludes non-state actors (e.g., NGOs, transnational corporations, influential individuals), thereby neglecting decentralized mechanisms of image-shaping. Finally, the findings are contextually bounded to interstate wars (e.g., the Russo-Ukrainian War); their applicability to different types of conflict, such as asymmetric international wars or proxy wars (which are by definition international), as well as civil wars---where insurgent narratives or external interventions may alter dynamics---remains untested and warrants specific investigation.

### Future directions

The limitations and findings of this scoping review reveal critical gaps that demand targeted investigation.

First, we acknowledge that our review, in practice, primarily examined interstate wars (e.g., the Russo-Ukrainian war)—that is, conflicts primarily between two or more recognized nation-states. While asymmetric wars (involving state and non-state actors of disproportionate power) and proxy wars (where states fight through third-party actors) are indeed salient forms of internationalized conflict, they present distinct dynamics in image formation that were not comprehensively captured in our current analysis due to our initial search and scope parameters. Future studies should, therefore, conduct structured comparisons between these subtypes. For instance, researchers could contrast the efficacy of historical “resistance narratives” in an interstate war (e.g., Ukraine’s WWII framing) versus their appropriation by non-state actors in a proxy war (e.g., Syrian opposition groups). This would clarify whether the diplomatic credibility constraints and leadership dynamics we identified in state-centric contexts persist when conflict agency is fragmented and outsourced.

Second, the Western-centric skew in extant literature obscures culturally embedded interpretations of image-shaping mechanisms. To address this, we suggest that researchers should develop mixed-methods frameworks analyzing non-Western media discourse (e.g., India’s The Hindu or Brazil’s Folha) to quantify how historical legacies like colonial trauma or anti-imperialism reframe perceptions of belligerents (cf. Western “democracy vs. autocracy” framing).

Third, despite recognizing non-state entities as emerging image agents, we note that no reviewed study systematically mapped their influence pathways. Future work should track real-time narrative battles between state and non-state actors (e.g., how Ukrainian diaspora TikTok campaigns #StopRussia challenged Kremlin diplomacy) using computational methods like dynamic network analysis.

Finally, we recommend methodological innovations should overcome the static snapshot limitation of current evidence. Process-tracing designs could model how diplomatic pivots (e.g., Turkey’s NATO stance shifts) dynamically mediate historical burdens during prolonged conflicts. Similarly, longitudinal text analysis of social media could quantify the decay rate of historical analogies (e.g., declining salience of “Nazi comparisons” as wars evolve).

The current scoping review sets a strong platform for future studies. By investigating proposed future avenues for studies, scholars can deepen their comprehension of factors shaping countries’ global image during times of war. The proposed avenues will serve to inform policymakers, media practitioners, and academic researchers in developing effective strategies for shaping international perception during and post-confrontation, thereby creating more peaceful and stable international relations.

## Supporting information

S1 Checklist Preferred Reporting Items for Systematic reviews and Meta-Analyses extension for Scoping Reviews (PRISMA-ScR) Checklist. (DOCX)

S1 Appendix Coding Book. (DOCX)
