# Supplementary material for: Global image of countries in international wars: A scoping review of influencing factors
Source: PLoS One. 2025 Oct 8;20(10):e0334095. doi: 10.1371/journal.pone.0334095 (PMC12507244; doi:10.1371/journal.pone.0334095)
Supplement: S1 Appendix — (DOCX) [file pone.0334095.s002.docx]

APPENDIX A

**Coding Book**

Coder MUST study this coding book thoroughly PRIOR to Coding Activity. The title of the scoping review is “**Global Image of Countries in International Wars: A Scoping Review of Influencing Factors**”

**A. INTRODUCTION:**

This codebook operationalizes the categorization framework for identifying factors influencing a country's international image during international wars.

**B. INSTRUCTIONS:**

All coders are required to study this coding book methodically prior to analyzing news stories. Coders are reminded to rigidly follow all the specified instructions in this coding book. Coders are also urged to consistently refer back to this coding book for technical clarification and guidance throughout the coding process.

Coders are required to FIRST identify the potential literature pertaining to the topics under study by paying attention to their (1). Headlines, (2). Abstract, (3). Keywords.

**C. CLASSIFICATION LOGIC AND RULES:**

| Approach | Procedure |
| --- | --- |
| Deductive | Three core categories (e.g., Historical, Diplomatic, Leadership) predefined based on preliminary literature synthesis. |
| Inductive | New subcategories (e.g., Digital Technology) added during full-text review, requiring independent identification by ≥2 reviewers and consensus. |
| Finalization | Contested items resolved by third reviewer arbitration. |

**I. CORE FACTOR DEFINITIONS AND EXAMPLES:**

| Factor | Definition | Example |
| --- | --- | --- |
| Historical | Invocation of historical events, collective memory, colonial legacies, or historical narrative frameworks to interpret current conflict behavior or moral standing. | 1. Historical war/conflict analogies (e.g., "Russian actions likened to Nazi aggression") 2. References to historical trauma/glory (e.g., "Ukraine's Holodomor memory") 3. Using history to assess national credibility (e.g., "Germany's pacifist image post-WWII") |
| Diplomatic | State-driven narrative shaping through strategic communication, alliance building, public diplomacy, or crisis management. | 1. Narrative construction (e.g., "Qatar used Al Jazeera to propagate claims") 2. Alliance signaling (e.g., "Ukraine sought NATO support") 3. Soft power/humanitarian diplomacy (e.g., "Turkey's humanitarian response to Syrian refugees") |
| Leadership | Leaders embodying national stances through symbolic acts, strategic communication, or crisis decision-making. | 1. Symbolic representation (e.g., "Zelensky as resistance icon") 2. Direct media engagement (e.g., "Biden framed Russo-Ukrainian War as democracy vs. autocracy") 3. Decisive actions (e.g., "Organizing refugee aid to demonstrate resolve") |

**II. ADDITIONAL FACTORS: DEFINITIONS AND EXAMPLES:**

| Factor | Definition | Example |
| --- | --- | --- |
| Geopolitical | Geopolitical position/alliances influencing conflict interpretation. | "Ukraine's EU-neighbor status shaped Western sympathy" |
| Military | Military conduct/outcomes directly impacting image. | "Violations of international humanitarian law incurred reputational costs" |
| Cultural | Cultural proximity/soft power affecting narrative reception. | "Cultural proximity influenced Western media framing of the Iraq War" |
| Governmental | Regime type (democratic/authoritarian) shaping credibility perceptions. | "Anti-democratic actions damaged Israel's international image" |
| Others | Factors not fitting initial categories were allowed to emerge.New codes were discussed, defined, and added to the codebook iteratively. | |

**III. QUALITY CONTROL IN CODING PROCESS:**

1)Calibration Training: Reviewers collectively coded 6 sample articles.

2)Blinded Dual-Extraction: Two reviewers independently marked factor-related passages in full texts.

3) Reliability Verification: Cohen’s κ was calculated for 10% randomly selected articles.

**D. RECORD:**

After the potential literatures are identified, coders are then required to attentively read the whole article and examine the relevant elements as specified in the coding book. Coders are required to record their observations/ analysis on the coding sheet.
